# Estradiol Fluctuation, Sensitivity to Stress, and Depressive Symptoms in the Menopause Transition: A Pilot Study

**DOI:** 10.3389/fpsyg.2019.01319

**Published:** 2019-06-12

**Authors:** Jennifer L. Gordon, Alexis Peltier, Julia A. Grummisch, Laurie Sykes Tottenham

**Affiliations:** Department of Psychology, University of Regina, Regina, SK, Canada

**Keywords:** menopause transition, perimenopausal depression, estradiol, trier social stress test, estrone-3-glucuronide

## Abstract

The menopause transition is associated with an increased risk of depressed mood. Preliminary evidence suggests that increased sensitivity to psychosocial stress, triggered by exaggerated perimenopausal estradiol fluctuation, may play a role. However, accurately quantifying estradiol fluctuation while minimizing participant burden has posed a methodological challenge in the field. The current pilot project aimed to test the feasibility of capturing perimenopausal estradiol fluctuation via 12 weekly measurements of estrone-3-glucuronide (E1G), a urinary metabolite of estradiol, using participant-collected urine samples in 15 euthymic perimenopausal women ages 45–55 years. Furthermore, it aimed to correlate E1G fluctuation (standard deviation across the 12 E1G measurements) with weekly mood and cardiovascular, salivary cortisol, and subjective emotional responses to the Trier Social Stress Test (TSST) at weeks 4, 8, and 12. Protocol acceptability and adherence was high; furthermore, E1G fluctuation was positively associated with anhedonic depressive symptoms and weekly negative affect. E1G fluctuation was also associated with increased heart rate throughout the TSST as well as higher levels of rejection, anger, and sadness. E1G fluctuation was not significantly associated with TSST blood pressure or cortisol levels. This study suggests a feasible method of assessing estradiol fluctuation in the menopause transition and provides support for the hypothesis that perimenopausal estradiol fluctuation increases sensitivity to psychosocial stress and vulnerability to depressed mood.

## Introduction

The menopause transition (a.k.a. *perimenopause*) represents the reproductive stage transitioning from regular menstrual cycles through the loss of ovulatory function and to the complete cessation of menses. The latter marks the onset of menopause. Between ages 42 and 55, nearly all women experience the menopause transition, which, on average, extends 5–6 years preceding the last menstrual period (Treloar, 1981; Oldenhave et al., 1993; Avis and McKinlay, 1995). A recent review article identified 12 cross-sectional studies comparing rates of elevated depressive symptoms in pre- and peri-menopausal women and concluded that 45–68% of perimenopausal women, versus only 28–31% of premenopausal women, report clinically significant elevations in depressive symptoms (Maki et al., 2019). Rates of *diagnosed* perimenopausal Major Depressive Disorder based on DSM-IV criteria (American Psychiatric Association, 2000) range between 12 and 23% (Cohen et al., 2006; Bromberger et al., 2011). Lost work productivity and medical costs associated with perimenopausal depression are estimated at $10,000 USD/woman/year (daCosta DiBonaventura et al., 2012).

Despite the substantial burden that perimenopausal depression places on millions of women, little is known about the biological mechanisms underlying its etiology. However, it has been hypothesized that the hormonal environment characterizing the menopause transition may play a role (Schmidt and Rubinow, 2009; Freeman, 2010). As a woman progresses through the menopause transition, menstruation becomes increasingly unpredictable and ovulation becomes increasingly rare – at the endocrine level, levels of progesterone, which rise following ovulation, become progressively more stable. In contrast, research comparing daily hormone levels in reproductive-aged and perimenopausal women have confirmed that the menopause transition is characterized by more extreme levels of estradiol (E2) than would be seen in a typical menstrual cycle; for example, luteal phase E2 levels have been shown to be higher in the menopause transition, sometimes reaching levels that are even double those generally seen in the late follicular phase (Hale et al., 2007; Hale and Burger, 2009). Furthermore, E2 levels in the early follicular phase have been shown to reach lower levels than typically observed in reproductive-aged women (Shideler et al., 1989). Several factors are believed to contribute to these more extreme E2 levels, including extreme fluctuation in follicle stimulating hormone, which controls the development of E2-producing follicles (i.e., eggs) in the ovaries, and greater variability in the number of follicles available for stimulation (O’Connor et al., 2003; Santoro and Randolph, 2011; Hale et al., 2014). It is theorized that increased E2 fluctuation – that is, repeated exposure to the rapid shift between the above-mentioned lower E2 “lows” and higher E2 “highs” than typically seen among reproductive-aged women – may play a key role in the etiology of perimenopausal depression (Gordon et al., 2015). Such a hypothesis would be consistent with the findings of a recent trial in which 172 euthymic perimenopausal women were randomized to receive either an E2 patch (0.1 mg), which would serve to reduce E2 fluctuation, or a placebo patch, for 12 months. Overall, women assigned to placebo were more likely to develop clinically significant depressive symptoms [score ≥16 on the Center for Epidemiologic Studies – Depression Scale (CES-D)] when compared to women assigned to E2 (odds ratio = 2.5).

To our knowledge, six studies have directly examined the relationship between natural fluctuations in E2 levels and mood in perimenopausal women. However, three of the six studies measured E2 levels less than once per year, likely contributing to their null findings (Avis et al., 2001; Woods et al., 2008; Bromberger et al., 2011). The fourth study, the Penn Ovarian Aging Study (Freeman et al., 2006) of 231 mid-life women, measured E2 levels twice per year, 1 month apart, over 8 years, and calculated E2 variability as the standard deviation associated with the two E2 measurements. In that study, years characterized by greater variability in E2 were associated with an increased risk of developing clinical elevations in depressive symptoms and major depressive disorder. The two most recent studies (Gordon et al., 2016a, 2016b) measured E2 with greater frequency, therefore providing a more direct test of whether it is E2 fluctuation rather than another epiphenomenon of menstrual irregularity that triggers perimenopausal depressive symptoms. The first study (Gordon et al., 2016b), examined the relationship between depressive symptoms and perimenopausal E2 fluctuation using four blood samples over the course of 14 months and found that the standard deviation across the four E2 measurements was positively associated with depressive symptoms at the end of the study among 20 women who had recently experienced a stressful life event. In the second study (Gordon et al., 2016a), salivary E2 levels and mood were concurrently assessed once weekly for four weeks among 30 perimenopausal women. The results of this pilot study revealed that greater change in E2 from one week to the next – particularly a greater increase in E2 – was associated with a subsequent increase in overall depressive symptoms, sadness, hopelessness, guilt, anger, anxiety, and feelings of social rejection. These results, in combination with the previously described studies, may suggest that greater mood sensitivity to acute changes in E2 is involved in the development of perimenopausal depression.

**FIGURE 1:**
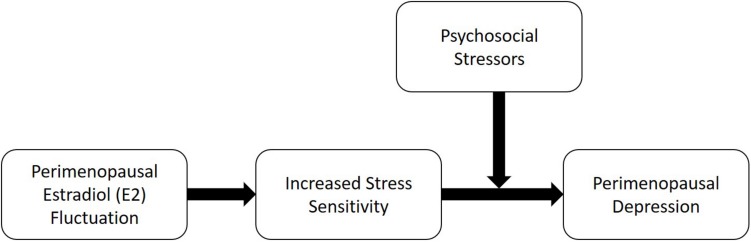
Theoretical model of perimenopausal depression development.

While the mechanisms by which E2 fluctuation may increase the risk of perimenopausal depression is unknown, increased sensitivity to psychosocial stress has been proposed as a possibility (Gordon et al., 2015). In one of the above-mentioned studies finding a significant relationship between E2 flux over 14 months and the emergence of perimenopausal depressive symptoms among women reporting at least one very stressful life event at baseline (Gordon et al., 2016b), E2 flux also predicted increased negative emotional responses to a standardized psychosocial stressor battery – the Trier Social Stress Test (TSST) – particularly exaggerated feelings of anger and rejection. These findings may suggest that increased E2 fluctuation in the menopause transition increases women’s sensitivity to stress; when this increased sensitivity is combined with stressful life events, depression ensues (Figure 1).

Indeed, the concept that increased sensitivity to stress might contribute to a vulnerability to developing perimenopausal depression is consistent with the broader literature suggesting that increased stress sensitivity may precede and contribute to risk for major depressive disorder unrelated to reproductive events (Pariante and Lightman, 2008). For example, hypercortisolism has been shown to precede the onset of first-episode major depressive disorder in high-risk adolescents (Goodyer et al., 2000). Furthermore, dysregulation of the hypothalamic-pituitary-adrenal (HPA) axis following successful depression treatment has been shown to predict relapse (Appelhof et al., 2006). The observation that euthymic relatives of individuals with a history of depression exhibit hypercortisolism (Mannie et al., 2007) further contributes to the view that stress axis dysregulation may be a risk factor for depression rather than simply a consequence or epiphenomenon of depression. However, while compelling, this hypothesized model of perimenopausal depression development requires additional testing.

### Rationale for the Current Study

Further research is needed to clarify the role that perimenopausal hormonal fluctuation and increased stress sensitivity play in triggering perimenopausal depressive symptoms as well as the ways in which these variables interact with the psychosocial environment to which a woman is exposed to predict depression risk. However, measuring reproductive hormone levels with sufficient frequency to adequately capture E2 fluctuation while minimizing participant burden and dropout presents an important methodological challenge, likely explaining why studies to date have assessed E2 levels and mood a maximum of four times. Plasma or serum E2 levels require repeated in-clinic venipuncture, making this a painful, and burdensome option for participants. While measuring E2 in participant-collected saliva samples is considerably more convenient, there is evidence suggesting that salivary E2 correlates only modestly with E2 levels in blood (Shirtcliff et al., 2000), particularly when used to detect levels that are as low as those sometimes seen in perimenopausal women (Tivis et al., 2005). Furthermore, E2 stability in saliva is relatively low and prone to deterioration with repeated freeze-thaw cycles (Lewis, 2006), making it important to maintain freezing temperatures when transporting saliva from participants’ homes. The current pilot study therefore aimed to assess the feasibility of capturing perimenopausal E2 fluctuation using a urinary metabolite of E2, estrone-3-glucuronide (E1G) – in 12 weekly participant-collected urine samples. Pregnanediol glucuronide (Pdg), a urinary metabolite of progesterone, was also measured to be included as a covariate in all analyses. These metabolites have been shown to correlate very highly (rs = 0.93–0.97) with serum levels of E2 and progesterone measured 1 day prior to urine collection (O’Connor et al., 2003). In other words, first-morning urine levels of E1G and PdG reflect an integrated measure of the overall hormone levels from the previous day. Furthermore, because urine can be non-invasively collected by participants at home, it represents an attractive alternative to blood.

A second goal of the current pilot study was to examine whether E2 fluctuation, measured using the above-mentioned methodology, would be associated with responses to a psychological laboratory stressor and/or a failure to habituate to such a stressor, administered multiple times. Thus, in addition to measuring weekly mood for 12 weeks, the current study administered the TSST – a highly structured and well-validated psychosocial stress protocol (Kirschbaum et al., 1993; Allen et al., 2014) – at weeks 4, 8, and 12. It was hypothesized that women exhibiting greater E1G fluctuation over the course of the 12 weeks would exhibit greater physiological and negative emotional responses to the TSST as well as demonstrate a failure to habituate to repeated administrations of the TSST. In addition, it was hypothesized that within-person analyses would reveal a significant relationship between greater weekly absolute change in E2 and both negative mood and greater responses to the TSST.

## Materials and Methods

### Participants

Fifteen medically healthy women were recruited who were aged 45–55 years and perimenopausal according to the Stages of Reproductive Aging Workshop (STRAW +10) criteria (early perimenopause, defined as menstrual cycle length 7+ days longer than usual; late perimenopause, defined as ≥2 skipped cycles and an interval of amenorrhea ≥60 days but within 1 year of last menstrual period) (Harlow et al., 2012). Exclusion criteria included the following: depressive symptoms in the clinically significant range, as defined as a CES-D score of 16 or above (Radloff, 1977; Thomas et al., 2001), currently using medications affecting mood or ovarian hormone levels (e.g., antidepressants and oral contraceptives), pregnant, or nursing. To ensure safety during stress testing, participants could not have a diagnosis of cardiovascular disease or hypertension or a resting blood pressure >140/90 at enrollment.

The study was advertised through flyers posted throughout Regina as well as through advertisements on social media. Participants were compensated $220 for completing the study. This research project was reviewed and approved by the University of Regina Research Ethics Board.

### Study Overview

**FIGURE 2:**
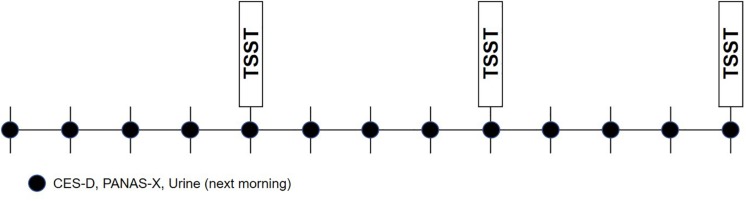
Study design.

Participants first underwent an enrollment visit during which their study eligibility was determined and informed written consent was obtained. At this time, participants completed questionnaires assessing detailed medical and medication history, demographic characteristics, and depressive symptoms. If determined eligible for the study, urine collection supplies were given to the participant to take home and detailed instructions on urine collection were given. Once weekly for 12 weeks, participants were emailed online mood surveys, and were reminded to collect a first-morning sample of urine the following day. On weeks 4, 8, and 12, on the day of mood survey completion (the day prior to urine collection), participants attended an in-person stress testing session in the laboratory during which emotion ratings, cortisol, heart rate, and blood pressure were assessed. On stress testing days, it was ensured that mood surveys were completed prior to stress testing. Figure 2 depicts the overall study design.

#### Weekly Mood and Hormone Measurement

Depressive symptoms were assessed once weekly using the Center for Epidemiologic Studies- Depression Scale (CES-D), a 20-item self-report form that asks about the frequency of depressive symptoms during the previous week on a 4-point scale of 0 (rarely) to 3 (most or all of the time) (Radloff, 1977). A score of 16 or above is commonly used as a cut-off for identifying potential clinical depression (Boyd et al., 1982) and is predictive of major depression (Thomas et al., 2001). Three subscales of the CES-D – somatic symptoms (items 1, 2, 5, 7, 11, and 20), negative affect (items 3, 6, 14, and 18) and anhedonia (items 4, 8, 12, and 16) (Carleton et al., 2013) – were also examined in the current study. The CES-D has been frequently used in perimenopausal samples (Avis and McKinlay, 1995; Daly et al., 2003; Freeman et al., 2006; Woods et al., 2008; Bromberger et al., 2011).

Positive and negative affect was also evaluated using the PANAS-X (Positive and Negative Affect Schedule – Expanded Form) (Watson and Clark, 1999). Participants rated the extent to which they endorse 60 emotions *right now* on a 5-point Likert scale, one being “very slightly or not at all” and five being “extremely.” The PANAS-X is one of the most widely used instruments in mood research; its validity and reliability as a measure of positive and negative affect have been well-established through rigorous statistical and time-spanning tests (Bagozzi, 1993).

Because first-morning voided urine levels of E1G and PdG reflect an integrated measure of the overall hormone levels from the previous day (O’Connor et al., 2003), urine collection occurred on the morning following the assessment of mood. On urine collection days, participants used the provided supplies to collect a sample of their first-morning voided urine in a plastic cup and used a syringe to fill one 2-ml polypropylene tube, which they placed in their home freezer in a tube storage box. This protocol was repeated on the same day every week for 12 weeks.

Once all urine samples had been collected by the participants, they were retrieved by a research assistant and taken to the laboratory at the University of Regina. Because urinary E1G and PdG concentrations are not affected by the repeated freezing and thawing of specimens (O’Connor et al., 2003), an icepack was used to simply keep the samples cool as they were taken to the laboratory within 2 h of pick up. Once received, samples were frozen at −40°C until they were assayed, which occurred within 45 days of receiving them.

#### Hormonal Assays

Estrone-3-glucuronide, a urinary metabolite of estradiol, was assayed using an enzyme immunoassay (Arbor Assays, Ann Arbor, MI, United States), with sensitivity at <22.5 pg/ml. The specificity is high, showing ≤0.1% cross-reactivity with similarly, structured compounds. Its cross-reactivity with estradiol is somewhat higher, however, at 5%. The intraassay coefficient of variation was 5.1% and the interassay coefficient of variation was 14.8%. PdG was also assayed using an enzyme immunoassay (Arbor Assays, Ann Arbor, MI, United States), with sensitivity at <0.180 ng/ml. The specificity is high, showing ≤0.2% cross-reactivity with similarly, structured compounds. Its cross-reactivity with 20-α-hydroxyprogesterone and 20-β-hydroxyprogesterone is somewhat higher, however, at 45 and 3.2%, respectively. However, the concentration of these compounds were expected to be excessively small in the samples. The intraassay coefficient of variation was 9.1% and the interassay coefficient of variation was also 9.1%. To account for differences in urine concentration, E1G, and PdG levels were adjusted for specific gravity using the formula recommended by O’Connor et al. (2003).

#### The Trier Social Stress Test (TSST)

At study weeks 4, 8 and 12, participants came to the laboratory to undergo the TSST, which has been shown to induce a reliable stress response (Kirschbaum et al., 1993). All laboratory sessions began between 2:00 pm and 5:00 pm to minimize the effect of diurnal changes in cortisol (Allen et al., 2014) and all three sessions were booked at the same time for each participant. Each session began with a 30-min rest period during which participants read magazines in a quiet room. The TSST involved four components:

(1) Pre-task instructions (1 min): participants were introduced to the committee who later listened to their speech and were given instructions for the mental arithmetic task;
(2) Speech preparation period (3 min): participants prepared their speech while the selection committee stood in the room;
(3) Speech (5 min): immediately following the preparation period, the selection committee asked the participant to deliver her speech. If the participant ended her speech before 5 min, the selection committee questioned her in a systematic fashion to ensure she spoke the entire 5 min; and
(4) Serial subtraction task (5 min): a 1-digit number was subtracted from a 4-digit number as fast and as accurately as possible for 5 min. For each mistake, the participant was instructed by a member of the selection committee to restart from the beginning.

Participants were video-recorded throughout their performance. To minimize habituation to this task, the exact speech topic, and instructions differed for each of the three laboratory stress sessions (job interview, promotion, and award nomination), as did the numbers involved in the mental arithmetic task, the location of the stress task, and the researcher administering the test.

#### Mood Measurements During the TSST

As recommended by Hellhammer and Schubert (2012), participants were asked to complete brief emotion rating scales every 10 min during the baseline period, following the speech task instructions, following the speech task, following the serial subtraction task, and every 10 min of the recovery period of the laboratory stress session. Specific emotions assessed included stress, sadness, anger, and feelings of rejection. The scale is anchored with 0 being “not at all” and 10 being “extremely.”

#### Physiological Measurements During the TSST

Blood pressure and heart rate were obtained at: minutes 20, 22, 24, 26, and 28 of the baseline period; minutes 0 and 2 of the speech preparation period, the speech, and the arithmetic task; and at minutes 2, 4, 8, 10, 13, 16, 20, 25, 30, 40, 45, 56, and 60 of the recovery period. These measures were then averaged to obtain a mean baseline, preparation, speech task, arithmetic task, and recovery value for each measure.

Saliva samples were collected for cortisol measurement at the end of the 30-min baseline rest period as well as minutes 0, 15, and 60 of the recovery period, aimed at capturing the peak in cortisol, which occurs 20–30 min after stressor onset (Allen et al., 2014). Salivary cortisol was determined using a Cortisol Enzyme Immunoassay Kit (Salimetrics) processed at the University of Regina SPIT Laboratory. Intra- and inter-assay coefficients of variation were low at 5.0 and 2.9%, respectively. The minimum cortisol detection level with this assay is 0.007 ug/dl.

### Data Management and Analysis

PROC MIXED in SAS 9.4 was used to carry out two sets of analyses – the first examining the within-person effect of weekly E1G fluctuation and the second examining the between-person effect of E1G fluctuation across all 12 weeks. In both cases, models were fitted using a restricted maximum likelihood (REML) estimation method, which is well-suited for small samples (Peugh, 2010). A first-order autoregressive covariance structure for within-person error was applied and the Kenward-Rogers correction was used to calculate the appropriate degrees of freedom.

For analyses testing the within-person effect of E1G fluctuation on weekly mood and stress test outcomes, the following fixed factors were included in the regression model: (1) absolute-value change in E1G since the previous week; (2) the direction of the change in E1G since the previous week; and (3) the interaction between these two variables. In addition, E1G and PdG levels on the day of outcome measurement (measured in urine the day after outcome measurement) were included as covariates.

For analyses testing the between-person effect of E1G fluctuation, the standard deviation in E1G across the 12 weekly measurements was examined in relation to weekly mood as well as responses to all three administrations of the TSST. Again, E1G and PdG levels on the day of outcome measurement (measured in urine the day after outcome measurement) were included as covariates.

For between-subject analyses examining stress testing outcomes, an additional model tested the interaction between the standard deviation in E1G and stress testing week (4, 8, or 12) to evaluate whether E1G fluctuation predicted habituation to repeated administrations of the TSST.

All estimates reported throughout the manuscript reflect the quantity of change in the dependent variable associated with 1 standard deviation’s worth of change in the independent variable.

## Results

### Participant Characteristics

The reproductive and hormonal characteristics of the 15 study participants are presented in Table 1. All but one woman was Caucasian and all were high school graduates, with 6/15 having a university degree. The mean gross household income was $90,000–112,999. The ages ranged from 45 to 54 years. All participants scored below 16 on the CES-D, with baseline scores ranging from 2 to 13.

**TABLE 1:** Reproductive and hormonal participant characteristics.

**Age (years)**	**Perimenopausal stage**	**# Months since LMP**	**Minimum E1G (pg/ml)**	**Maximum E1G (pg/ml)**	**E1G SD (pg/ml)**	**Minimum PdG (pg/ml)**	**Maximum PdG (pg/ml)**
52	Late	3	1,115	4,577	1,129	27	180
52	Late	1	3,171	9,501	2,012	143	803
53	Late	0	3,511	11,640	2,324	233	848
46	Late	5	3,946	11,940	2,399	249	663
46	Late	5	341	9,138	2,866	26	842
54	Late	10	5,487	16,840	3,378	175	874
46	Late	4	3,655	35,920	8,954	111	444
47	Early	0	2,891	42,830	13,334	124	2,315
50	Late	4	2,802	54,690	15,551	94	1,940
46	Early	1	14,970	61,350	15,815	231	5,750
44	Late	5	4,167	93,910	28,082	240	4,723
47	Late	2	7,747	122,200	35,568	634	3,757
45	Early	0	10,970	133,100	41,181	389	8,320
50	Late	0	5,188	139,300	43,456	94	987
47	Late	1	5,826	191,800	55,972	216	5,074

*Note: LMP: last menstrual period.*

Estrone-3-glucuronide fluctuation across the 12-week study (calculated as the standard deviation in E1G levels) was significantly correlated with a participant’s maximum E1G level [r(15) = 0.99, *p* < 0.0001], mean E1G level [r(15) = 0.97, *p* < 0.0001], maximum PdG level reached [r(15) = 0.68, *p* = 0.006], and mean PdG [r(15) = 0.56, *p* = 0.032]. However, E1G fluctuation was not correlated with either minimum E1G [r(15) = 0.43, *p* = 0.104] or minimum PdG level reached [r(15) = 0.44, *p* = 0.101].

### Protocol Compliance

Overall protocol adherence was high: participants completed 100% of the required weekly urine samples and 84% (10 out of 12) of the weekly mood surveys. In three instances, the day on which the weekly urine sample and mood survey occurred were changed to accommodate participants’ schedules (e.g., being out of town for a part of the week). All but one participant completed all three stress testing sessions; this participant was withdrawn from the stress testing portion of the study after her blood pressure reached concerningly high levels in the first session. Her blood pressure values from the first stress testing session were not included in any analyses.

### Overall Efficacy of the Stress Protocol

**FIGURE 3:**
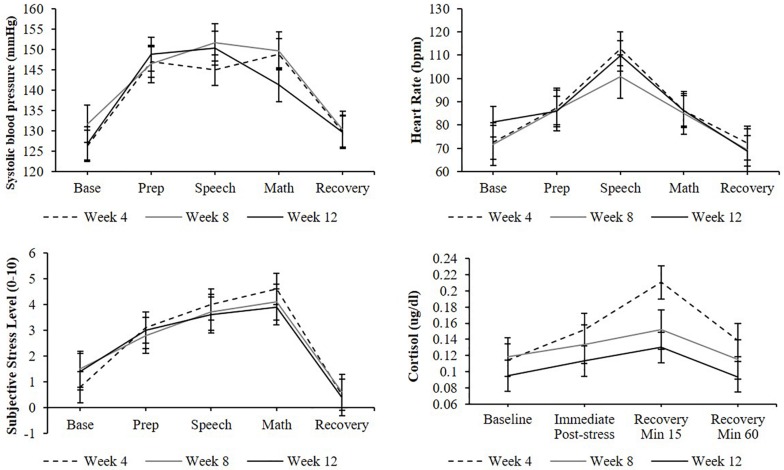
Cardiovascular, cortisol, subjective stress responses to stress testing, and administered at weeks 4, 8, and 12. Standard error bars shown.

A significant effect of stress testing phase was found for systolic blood pressure [*F*(4,136) = 30.9, *p* < 0.0001], diastolic blood pressure [*F*(4,127) = 32.5, *p* < 0.0001], heart rate [*F*(4,77) = 7.9, *p* < 0.001], and subjective stress levels [*F*(6,223) = 28.1, *p* < 0.0001] such that levels during the preparation, speech, and arithmetic phases were significantly higher than baseline levels (*p*s < 0.05; Figure 3). Similarly, a significant effect of stress testing phase was found for cortisol [*F*(3,106) = 8.5, *p* < 0.0001] such that both the second and third samples (but not the fourth) were significantly greater than the baseline sample (*p*s < 0.05). Non-significant effects of TSST administration number (*p*s > 0.05) and non-significant phase-by-administration number interaction (*p*s > 0.05) indicate that participants demonstrated a similar cardiovascular, cortisol, and subjective stress response across all three TSST administrations.

### Within-Person Effects of E1G Fluctuation

Table 2 depicts the results of analyses investigating the within-person effect of E1G fluctuation on responses to the TSST and weekly mood, which included on-the-day E1G and PdG levels as covariates. The results suggest that change in E1G levels from one week to the next, regardless of the direction of the change, was associated with higher heart rate and anger in response to the TSST. In addition, a drop in E1G from one week to the next was associated with more negative affect and tended to be associated with a higher total CES-D score, a higher CES-D anhedonia subscale score, as well as higher diastolic blood pressure during the TSST. The effect of hormone levels were non-significant for the most part, with the exception of a positive effect of E1G and negative effect of PdG on anger.

**TABLE 2:** Within-person effects of weekly absolute value change in E1G, absolute value E1G change by change direction, E1G level, and PdG level on weekly mood and responses to the TSST.

**Variable**	**β(SEM) |ΔE1G| ^a^**	**β(SEM) |ΔE1G| X direction^b^**	**β(SEM) E1G level**	**β(SEM) PdG level**
**Physiological responses to the TSST**				
Heart rate	$3.0\,{\left(5.5\right)}^{*}$	-3.0⁢(6.3)	-1.4⁢(4.6)	2.0⁢(2.7)
Systolic blood pressure	-2.4⁢(2.4)	-2.4⁢(2.7)	2.0⁢(2.0)	0.3⁢(1.2)
Diastolic blood pressure	1.9⁢(2.1)	$-3.5\left(2.1\right){}^{\#}$	2.1⁢(1.6)	0.9⁢(0.9)
Cortisol AUC	1.1⁢(2.3)	-0.5⁢(0.5)	0.2⁢(1.9)	1.2⁢(1.1)
**Emotional responses to the TSST**				
Rejection	0.1⁢(0.1)	0.1⁢(0.1)	0.1⁢(0.1)	-0.1⁢(0.0)
Anger	$0.3\,{\left(0.1\right)}^{*}$	0.2⁢(0.1)	$0.4\,{\left(0.1\right)}^{**}$	$-0.3\,{\left(0.0\right)}^{**}$
Stress	0.1⁢(0.4)	-0.2⁢(0.4)	$-0.6\left(0.3\right){}^{\#}$	0.0⁢(0.2)
Sadness	0.0⁢(0.1)	-0.1⁢(0.1)	0.0⁢(0.1)	0.1⁢(0.0)
**Weekly mood**				
Negative affect	1.0⁢(0.7)	$-1.5\,{\left(0.7\right)}^{*}$	1.1⁢(0.7)	0.1⁢(0.7)
Positive affect	0.5⁢(1.0)	-0.3⁢(1.1)	1.0⁢(1.1)	0.2⁢(0.6)
CES-D total	0.5⁢(0.9)	$-1.9\left(1.9\right){}^{\#}$	0.9⁢(0.9)	0.1⁢(0.5)
CES-D negative affect	0.1⁢(0.2)	-0.3⁢(0.3)	0.2⁢(0.2)	0.1⁢(0.1)
CES-D anhedonia	0.2⁢(0.3)	$-0.7\left(0.4\right){}^{\#}$	0.6⁢(0.4)	-0.3⁢(0.2)
CES-D somatic	0.1⁢(0.3)	-0.1⁢(0.4)	-0.1⁢(0.4)	0.1⁢(0.2)

^a^Indicates the magnitude of the change in the dependent variable that is associated with 1 SD change in E1G (in either direction). ^b^Indicates the difference in the effect of 1 SD of change in E1G when the direction of the change from the previous week is up vs. down. A negative value indicates that the effect of a decline in E1G is stronger than an increase in E1G. ^#^*p* < 0.10; ^*^p < 0.05; ^∗∗^*p* < 0.01.

### Between-Person Effects of E1G Fluctuation on Responses to the TSST

#### Physiological Responses

**FIGURE 4:**
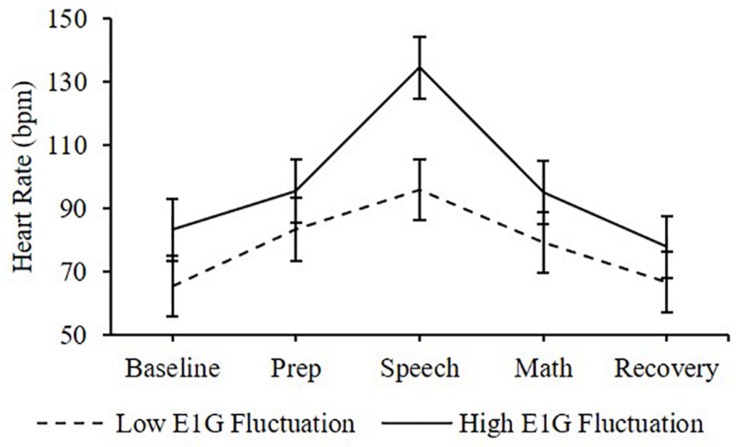
Mean heart rate during stress testing among women in the bottom vs. top tertile for mean E1G fluctuation, illustrating a significant relationship between continuous E1G fluctuation and heart rate (*p* = 0.020). Standard error bars shown.

**FIGURE 5:**
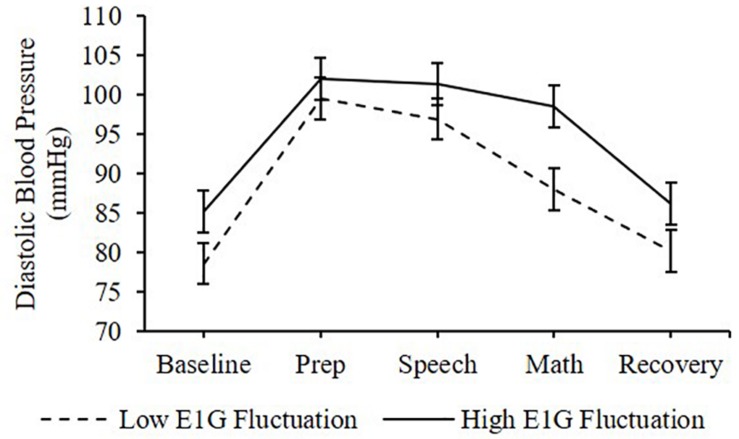
Mean diastolic blood pressure during stress testing among women in the bottom vs. top tertile for mean E1G fluctuation, illustrating a near-significant relationship between continuous E1G fluctuation and blood pressure (*p* = 0.056). Standard error bars shown.

Adjusting for E1G and PdG levels on the day of the TSST, E1G fluctuation across the twelve-week study was associated with greater heart rate [β(SE) = 6.6(2.9), *p* = 0.020] (Figure 4) and diastolic blood pressure [β(SE) = 1.8(1.0), *p* = 0.056] (Figure 5) but not systolic blood pressure [β(SE) = -0.4(1.2), *p* = 0.809] or cortisol [β(SE) = 0.0(0.0), *p* = 0.296] throughout stress testing. E1G fluctuation did not interact with stress testing phase (*p*s > 0.05) or week (*p*s > 0.05) to predict any cardiovascular variables or cortisol.

#### Emotional Responses

Adjusting for on-the-day E1G and PdG levels, greater E1G fluctuation over the entire 12 weeks predicted greater and overall feelings of rejection [β(SE) = 0.3(0.0), *p* < 0.001], anger [β(SE) = 0.3(0.0), *p* < 0.0001], but not stress [β(SE) = 0.3(0.2), *p* = 0.094] in response to the TSST. While there was also a significant relationship between E1G fluctuation and overall sadness [β(SE) = 0.3(0.0), *p* < 0.0001], a significant interaction between E1G fluctuation and stress testing phase (*p* < 0.0001) suggested that the effect of E1G fluctuation was only significant for the post-speech [β(SE) = 0.5(0.1), *p* < 0.001], and post-arithmetic [β(SE) = 0.7(0.1), *p* < 0.0001] assessments. E1G fluctuation did not otherwise interact with testing phase (*p*s > 0.05) and did not significantly interact with administration number (*p*s > 0.05) to predict any emotional responses to the TSST.

### Between-Person Effects of E1G Fluctuation on Weekly Mood

#### PANAS-X

**FIGURE 6:**
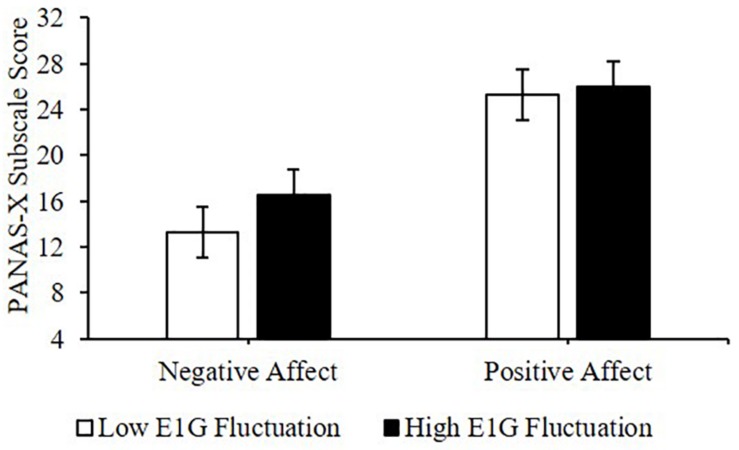
Mean PANAS-X subscale scores among women in the bottom vs. top tertile for mean E1G fluctuation, illustrating a significant relationship between continuous E1G fluctuation, and weekly negative affect (*p* = 0.042) but not sadness (*p* = 0.691).

Adjusting for E1G and PdG levels, greater E1G fluctuation over the entire 12 weeks, determined using the standard deviation in E1G levels, predicted greater weekly negative affect [β(SE) = 1.9(0.8), *p* = 0.042] but not positive affect [β(SE) = 0.7(1.8), *p* = 0.691] (Figure 6).

#### CES-D

**FIGURE 7:**
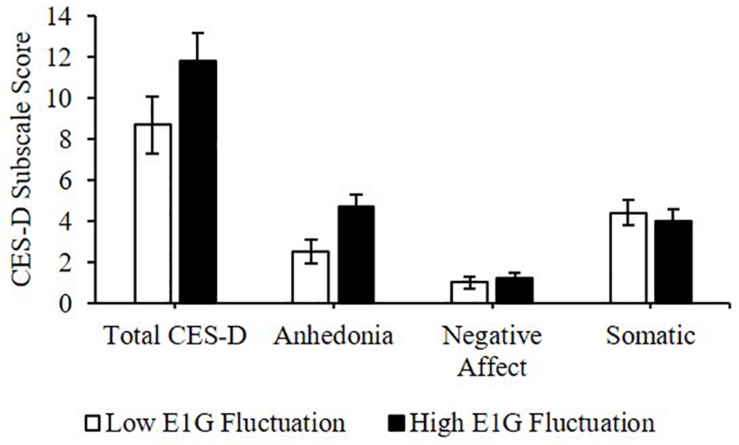
Mean CES-D subscale scores among women in the bottom vs. top tertile for mean E1G fluctuation, illustrating a significant relationship between continuous E1G fluctuation, and the anhedonia subscale of the CES-D (*p* = 0.016) as well as a weak trend between greater E1G fluctuation and a higher total CES-D score (*p* = 0.122). No effect of E1G fluctuation was seen on the somatic (*p* = 0.349) or the negative affect CES-D subscale (*p* = 0.548). Standard error bars shown.

Greater E1G fluctuation predicted higher scores on the anhedonia subscale of the CES-D [β(SE) = 0.8(0.3), *p* = 0.016] and a weak trend was seen between greater E1G fluctuation and a higher total CES-D score [β(SE) = 1.1(0.7), *p* = 0.122]. However, no effect of E1G fluctuation was seen on the somatic (*p* = 0.349) or the negative affect (*p* = 0.548) CES-D subscales (Figure 7).

## Discussion

The current study aimed to test the feasibility of capturing perimenopausal reproductive hormone flux using weekly urine samples for the measurement of estradiol and progesterone metabolites; furthermore, it examined the relationship between E1G fluctuation and mood (assessed using weekly questionnaires) and stress sensitivity (assessed using physiological and emotional responses to multiple administrations of the TSST). The feasibility of measuring reproductive hormone fluctuation using reproductive hormone metabolites was supported, as was the study protocol: participants were highly adherent to the weekly urine samples and mood surveys, and stress testing was successful in triggering a stress response, even upon the third administration. Results suggested that E1G fluctuation across the 12-week study was associated with more weekly negative affect and anhedonic depressive symptoms, as well as higher heart rate, diastolic blood pressure, and feelings of rejection, anger and sadness during the TSST. At a within-subjects level, greater change in E1G from one week to the next was associated with greater negative affect, as well as higher heart rate and anger during stress testing.

Reflecting on some of the methodological details that may have contributed to our study’s success, we suspect that participant reminders by email, phone, or text (depending on the participant’s preference) on the day prior to mood measurement and prior to urine collection were critical. Allowing participants to choose the day of the week that was most convenient for them and allowing flexibility for cases in which participants were going to be out of town was also helpful. Providing disposable cups and disposable syringes, allowing the participant to transfer urine from a large cup to the 2 ml polypropylene tube also seemed to contribute to participants’ willingness to comply with the urine collection protocol. Related to the repeated administration of the TSST: the fact that all three stress testing sessions were administered not only using different speech and arithmetic task instructions but also by a different person and in a different location each time may have helped to minimize habituation to the stressor despite repeated administrations within a short timespan. One final practical detail we wish to mention for any researcher considering incorporating the measurement of urinary metabolites in their research relates to the method used to adjust metabolite levels according to the concentration of the urine. One commonly used method for doing so involves measuring and adjusting for urinary levels of creatinine, a by-product of muscle activity that is excreted in urine (Taussey, 1954). Our decision to use specific gravity with a refractometer, which provides a proxy of urine concentration by measuring the absorption of light through the sample, was based on two considerations: (1) adjusting for specific gravity has been found to be as effective as creatinine correction, and even more effective in cases of very dilute or highly concentrated urine (Miller et al., 2004); (2) in the long run, specific gravity would be more cost-effective as it requires a one-time purchase of a refractometer whereas creatinine correction requires the repeated purchase of creatinine assay kits; and (3) specific gravity is much less time-intensive, requiring fewer human resources.

Despite the small sample size and limited statistical power of this pilot study, we detected a significant relationship between E1G fluctuation and negative mood, indicated by the negative affect subscale of the PANAS-X and the anhedonia subscale of the CES-D. Importantly, the effect of E1G fluctuation on mood was independent of hormone *levels*. This is consistent with previous studies linking estradiol fluctuation with perimenopausal mood (Freeman et al., 2006; Gordon et al., 2016a, 2016b) and consistent with the observation that depression risk decreases in the postmenopausal period (Freeman et al., 2004; Cohen et al., 2006; Bromberger et al., 2007, 2011; Woods et al., 2008), when estradiol levels are low but stable. Finally, it is consistent with the findings of a recent clinical trial comparing the efficacy of transdermal estradiol vs. placebo in preventing depressive symptoms in perimenopausal and early postmenopausal women (Gordon et al., 2018): in this trial, the benefits of estradiol were most apparent in the early perimenopausal women when compared to the late perimenopausal and early postmenopausal women. Since early perimenopausal women show the highest mean E2 levels but also the highest E2 fluctuation, these findings suggest that the beneficial effects of transdermal E2 were mediated by its E2 *stabilization* effects rather than by *increasing* E2. It is noteworthy that although all participants in the current study were in the menopause transition, the range of E1G fluctuation was considerable, suggesting large individual variability in the amount of ovulatory activity occurring over the 12-week study. Our findings therefore suggest that although the overall risk for depressive mood is increased in the menopause transition, periods of relatively less ovulatory activity do occur and are accompanied by less negative mood.

Between-subject E1G fluctuation was also associated with higher heart rate and diastolic blood pressure, as well as feelings of rejection, sadness, and anger in response to the TSST, consistent with the sole previous study examining the effect of estradiol fluctuation on responses to the TSST (Gordon et al., 2016b). However, the effect of E1G fluctuation appeared to be similar across all three stress test administrations, contrary to our hypothesis that women with greater E1G fluctuation would show less habituation than women experiencing less fluctuation. Furthermore, the fact that the effect of E1G fluctuation did not interact with stress testing phase to predict most outcomes (apart from sadness) raises the possibility that greater E1G fluctuation may be associated with greater general resting-state arousal, perhaps resulting from heightened negative mood, rather than an increased sensitivity to stress that contributes to an increased vulnerability to depression. Further research is needed to clarify whether this increased stress sensitivity is a mechanism mediating the relationship between estradiol fluctuation and perimenopausal depressive symptoms or whether it is a consequence of hormonally triggered depressive symptoms. Experimental research directly manipulating estradiol levels and examining its effects on stress reactivity would help clarify the direction of this relationship.

The mechanisms by which estradiol fluctuation may increase sensitivity to stress and risk for depressed mood remain to be clarified. Candidate mechanisms underlying the negative mood effects of acute drops in estradiol include withdrawal from estradiol’s anti-inflammatory (Vegeto et al., 2008), neuroprotective (Bredemann and McMahon, 2014), and serotonergic (Rubinow et al., 1998) effects. However, there is also evidence suggesting that a subset of the population – particularly women with current (Gordon et al., 2016a) or past (Jacobs et al., 2015) depression – may be especially sensitive to acute *increases* in estradiol. While the mechanisms underlying this effect are largely unknown, one postmortem study observing that women with major depressive disorder at the time of their deaths had lower estradiol receptor α expression in the frontal cortex and hippocampus suggests that the altered expression and distribution of estradiol receptors in limbic and frontal regions may be involved (Perlman et al., 2005). It has also been suggested that the effect of estradiol fluctuation on mood may be mediated by fluctuations in neurosteroids (steroids that are produced *de novo* in the brain) that are modulated by estradiol. For example, allopregnanolone is a progesterone-derived neurosteroid that exerts both anxiolytic (Bitran et al., 1995) and antidepressant (Rodrìguez-Landa et al., 2007) effects via its GABAergic effects and is positively modulated by estradiol (Bernardi et al., 2003; Pluchino et al., 2005, 2009). Research conducted in rodents suggests that large fluctuations in allopregnanolone can reverse its psychological effects such that it becomes anxiogenic rather than anxiolytic (Shen et al., 2007). The fact that estradiol fluctuation was not related to self-reported stress levels or cortisol in the current study does not fully support the involvement of this mechanism; however, it should not be ruled out given our limited statistical power.

In considering the mechanisms linking estradiol fluctuation with perimenopausal mood, it should be emphasized that it is likely that the processes involved in mediating estradiol’s effects on mood may vary from woman to woman, thus making individual women differentially sensitive to estradiol change in one direction or the other – this would be consistent with research observing a high degree of individual variability both in the magnitude and direction in sensitivity to reproductive hormone change across the menstrual cycle (Eisenlohr-Moul et al., 2016). The possibility that individual women may be differentially sensitive to changes in E2 – in both direction and magnitude – may help explain why many of the within-person effects of E1G fluctuation were found to be non-significant. The methods used in this pilot study, in a larger sample of women, may prove useful in examining individual differences in sensitivity to E2 change.

The current study findings should be interpreted in light of some limitations. First, the small sample size raises questions about the generalizability of the findings and limits our ability to examine the moderation of estradiol’s effect on mood, such as life stress or depression history. Second, although the use of once-weekly samples may be sufficient in capturing between-person effects of hormonal fluctuation on overall mood, it is an imperfect method for capturing the acute estradiol changes that can impact mood. Daily or every-other-day measurements may be better suited for such purposes; however, the risk of overburdening participants and therefore increasing participant non-adherence and dropout must be weighed against the advantages of measuring hormone levels with greater frequency. The BIMORA study is one study that successfully used daily urine samples – over five six-month collection intervals – to examine reproductive hormone trajectories across the menopause transition (Ferrell et al., 2005). However, it is noteworthy that only 35% of eligible women agreed to participate in BIMORA and study retention was only 63%, which may be particularly problematic in a study on perimenopausal depression since we would likely expect women to be less adherent in the context of elevated depressive symptoms. The protocol used in the current study may therefore be an acceptable middle ground that balances participant burden and the accuracy with which E2 fluctuation is measured. Third, excluding women with elevated depressive symptoms at baseline limits the range of the outcome variables assessed and may limit the generalizability of the current study’s findings to non-euthymic women. The inclusion of exclusively euthymic women may help explain why effects of E1G fluctuation were seen on the negative affect subscale of the PANAS but not of the CES-D, as the latter assesses more severe depressive mood. Finally, including premenopausal and postmenopausal women in the current study would have allowed us to compare mood and stress reactivity and their relation to hormonal fluctuation across the reproductive lifespan.

In conclusion, the current pilot study suggests a protocol that may be useful for investigating the role of estradiol fluctuation in the development of perimenopausal depressive symptoms. Furthermore, our findings suggest that periods of relative ovarian inactivity and estradiol stability in the menopause transition are associated with less self-reported negative mood and decreased sensitivity to psychosocial stress in a laboratory setting. Further research is needed to confirm whether perimenopausal depressive symptoms are more prone to waxing and waning than typical depression, depending on the hormonal environment to which a woman is being exposed at any given time.

## Ethics Statement

The protocol was approved by the University of Regina Research Ethics Board. All subjects gave written informed consent in accordance with the Declaration of Helsinki.

## Author Contributions

JeG, AP, and LST contributed to conception and design of the study. JeG and JuG performed the statistical analysis. JeG wrote the first draft of the manuscript. AP and JuG wrote sections of the manuscript. LST conducted the hormone assays. All authors contributed to manuscript revision, read, and approved the submitted version.

## Conflict of Interest Statement

The authors declare that the research was conducted in the absence of any commercial or financial relationships that could be construed as a potential conflict of interest.
